# A Systematic Review of Group Social Skills Interventions, and Meta-analysis of Outcomes, for Children with High Functioning ASD

**DOI:** 10.1007/s10803-018-3485-1

**Published:** 2018-02-08

**Authors:** J. Wolstencroft, L. Robinson, R. Srinivasan, E. Kerry, W. Mandy, D. Skuse

**Affiliations:** 1 The Great Ormond Street Institute of Child Health, University College London, 30 Guilford Street, London, WC1N 1EH UK; 2 Institute of Psychiatry, King’s College London, 103 Denmark Hill, London, SE5 8AF UK; 3 Division of Psychology and Language Sciences, Faculty of Brain Sciences, University College London, 1-19 Torrington Place, London, WC1E 6BT UK

**Keywords:** Social skills, Social competence, Social responsiveness scale

## Abstract

Group social skills interventions (GSSIs) are a commonly offered treatment for children with high functioning ASD. We critically evaluated GSSI randomised controlled trials for those aged 6–25 years. Our meta-analysis of outcomes emphasised internal validity, thus was restricted to trials that used the parent-report social responsiveness scale (SRS) or the social skills rating system (SSRS). Large positive effect sizes were found for the SRS total score, plus the social communication and restricted interests and repetitive behaviours subscales. The SSRS social skills subscale improved with moderate effect size. Moderator analysis of the SRS showed that GSSIs that include parent-groups, and are of greater duration or intensity, obtained larger effect sizes. We recommend future trials distinguish gains in children’s social knowledge from social performance.

## Introduction

The social difficulties in autism spectrum disorders (ASD) are characterized by deficits in social cognition, interaction and communication (American Psychiatric Association 2013). These deficits are often referred to collectively as *social skills* difficulties. The term *social skills* is a complex and multi-facetted construct.

### Definitional Issues

Many competing definitions and theoretical models of social skills exist (Elliott and Gresham 1987; Gresham 1986; Merrell and Gimpel 2014; Nangle et al. 2010), but the core features invariably include behaviours that are performed in a social context (McFall 1982) and entail person to person engagement (Cordier et al. 2015).

Social skills deficits are an important target for intervention because they have a significant impact on academic, adaptive and psychological functioning (Coie et al. 1995; Elliott et al. 2001; Spence 1995). Group social skills interventions (GSSIs) are often recommended for children with high functioning ASD. As their name indicates they aim to improve social skills, suggesting that well-designed programmes aim to improve both social performance and social knowledge. Their use has increased substantially in the last 15 years (Volkmar et al. 2004; Reichow and Volkmar 2010; Reichow et al. 2012; Kasari et al. 2012; Matson et al. 2007).

The content, teaching strategy, mode of delivery and intensity of therapy provided by GSSIs is variable. Manualised group GSSIs typically include behavioural modelling of a specific social skill, practising the skill through role-play and individualised feedback on performance. Some teaching strategies are ‘didactic’, with structured lessons. Others elicit social skills through play; these are called ‘performance’ interventions (Kaat and Lecavalier 2014). The mode of delivery differs between GSSIs, and can require a combination of parent, peer or teacher involvement. Some programmes are intense, requiring 12 or more 90 min sessions, delivered weekly. Others require attendance at summer camps.

### Effectiveness of GSSIs

Despite the popularity of GSSIs, evidence for their effectiveness is limited (Schneider 1992; Beelmann et al. 1994), in part because of weak study methodology (White et al. 2007; Cappadocia and Weiss 2011; Ferraioli and Harris 2011; Rao et al. 2008; Reichow and Volkmar 2010; McMahon et al. 2013). Objective analysis has been hindered because outcomes are often measured by just one mode (e.g. questionnaire or observation) and by a limited range of informants (often parents, and/or teachers). Both the choice of outcome measures and the choice of informants can influence expectancy biases and mask or exaggerate treatment effects (McMahon et al. 2013). Parents are the most commonly used informants, but their reports are prone to expectancy bias (McMahon et al. 2013). They may also find it difficult to characterise their child’s social limitations in comparison to other (typical) children (Schneider and Byrne 1989).

Besides parents, other potential sources of information about treatment effectiveness include ratings of outcomes by the participants themselves, the study’s own administrators, teachers, peers, study staff and blind observers. Teachers and blinded study administrative assessors can report on whether changes of performance generalise to other settings, outside the family (White et al. 2007; Gates et al. 2017). Self-report is particularly valuable to evaluate gains in social knowledge.

### Outcome Measures

Whilst blind-rated observations of behavioural change are potentially the most objective measures of outcome, questionnaires are used more frequently (Kaat and Lecavalier 2014). Questionnaires can yield biased data, for instance if rated by parents who are subject to expectancy effects. For that reason, they are sometimes combined with cognitive measures, behavioural observations and sociometric tasks (McMahon et al. 2013; Kaat and Lecavalier 2014). Each mode of reporting has advantages and disadvantages. Observations invariably encompass only a brief period of data collection, in limited environments, so may lack external validity unless repeated observations are obtained in different settings. In contrast, self-report of increases in knowledge and parental-reports of behavioural change, whilst reflecting broader environmental contexts, are both subject to positive expectancy biases. Teacher reports, whilst less subject to expectancy bias, may in contrast reflect a lack of sensitivity to real change, due to limited opportunities to identify social behaviour and potential problems associated with their interpretation and scoring of measures.

Gresham (1997) made a useful distinction between social skills *acquisition* deficits (an individual lacks the knowledge to perform a social behaviour) and social skills *performance* deficits (the individual has relevant skills knowledge but fails to apply that knowledge in real-life situations). There is evidence to support a theoretical distinction between social performance and social knowledge (Lerner and Mikami 2012; Lerner et al. 2012; Lerner and White 2015).

Several recent reports have conducted meta-analyses on the effectiveness of GSSIs (Gates et al. 2017; Reichow et al. 2012). Reichow et al. (2012) found evidence for modest improvements in social competence on both parent-report measures and self-report measures of friendships. Gates et al. (2017) found self-reports of knowledge acquisition were associated with large effect sizes in contrast to small effect sizes for parent and observer reports of performance (both blinded and non-blinded). Non-significant effects were observed for teacher reports. The self-report effect sizes appeared to be driven by increases in social knowledge rather than improvements in social performance (Gates et al. 2017). As indicated, a risk with participants rating themselves is that they tend to overestimate perceived improvements in their social skills (Gates et al. 2017; Kaat and Lecavalier 2014).

In this review, the assessment of social skills acquisition is focused on changes in *social performance* as measured by parental report, because the GSSIs meeting our criteria for inclusion had in common parent-rated outcomes. We acknowledge that a more complete account would include *social knowledge acquisition* (Gresham 1997) but the relevant data were lacking. Parents are the most frequently used informants. Among parent-rated measures employed by studies of GSSI effectivness, the social responsiveness scale (SRS) (Constantino and Gruber 2012) and the social skills rating system (SSRS) (Gresham and Elliott 1990) predominate (Crowe et al. 2011; Kaat and Lecavalier 2014; Matson and Wilkins 2009).

To date, GSSI reviews have assumed that diverse social skills outcome measures reflect the same underlying constructs, hence they have assumed that it is legitimate to combine the scores of a wide range of different tools for the purpose of outcome analysis (Reichow et al. 2012; Gates et al. 2017). As discussed, because social skills encompass distinct dimensions of, at least, social knowledge and social performance, this approach is not ideal (Kaat and Lecavalier 2014). We have taken advantage of the fact there are recently published well-designed studies on performance change using the same outcome measures (SRS and/or the SSRS), hence an opportunity to conduct a new meta-analysis with higher internal validity.

### Aims

In this review, we conducted a meta-analysis focussed on individual parent-report measures of outcome, with a focus on the degree to which change in SRS and/or SSRS scores is mediated by a GSSI.

There has been no systematic review of the GSSI teaching syllabus content (Koenig et al. 2009). Few manualised intervention programmes have been published, but it is thought that intervention-specific factors such as treatment duration, intensity, teaching strategy (e.g. didactic or performance) and parental involvement may moderate program success (Reichow et al. 2012; McMahon et al. 2013). We thus also aimed to evaluate whether intervention-specific factors such as type of parent group, method of delivery, or duration have a moderating impact on specific aspects of social knowledge or performance improvement, by means of moderation analysis.

We hypothesised that specific dimensions of social skills are responsive to specific aspects of GSSI, providing support for the relative strengths (and weaknesses) of different GSSI programmes.

## Methods

### Literature Search

Online electronic searches were conducted on the EMBASE, Medline (Ovid), PsycINFO and CINAHL databases in December 2016. Eligibility criteria included medical subject heading (MeSH) key terms including ‘social skills’ and ‘group interventions’, as well as filters for the age of participants (filters overlapping with a 6–25 years age range) and the language of publication (English language). The complete search strategy can be found in the supplementary materials. The reference lists of studies included in the electronic search were screened to identify additional studies.

### Inclusion and Exclusion Criteria

#### Systematic Review

Two independent reviewers (JW and EK) rated the abstracts against the eligibility criteria. Disagreements between reviewers were resolved through discussion. A third independent reviewer was available for further consultation if consensus could not be reached, but was not required. Published studies were eligible if they met the following criteria: (1) randomised control trials (RCT) using a delayed treatment control group (2) multi-modal group social skills intervention including two or more children delivered by professionals (3) participants aged 6–25 years (4) assessment of social skills using the SRS and/or SSRS (Box 1). Only RCTs employing a delayed treatment control group were retained to reduce heterogeneity and increase internal validity.

**Box 1:** Properties of the SRS and SSRS

The SRS and the SSRS are both norm-referenced questionnaires. They can be completed in 15–20 min. Both assessments predominantly focus on social performance. The SRS was designed to measure autistic traits quantitatively and the instrument has convergent validity with other ASD diagnostic tools (Constantino and Gruber 2012). The SSRS was designed to provide a comprehensive picture of social behaviour rather than specific ASD traits (Gresham and Elliott 1990). The SRS subscales comprise social awareness, social cognition, social communication, social motivation, and restricted interests and repetitive behaviour (RRB). The SSRS subscales examine social skills (including cooperation, assertion, self-control, responsibility) and problem behaviours (including externalising behaviours, internalising behaviours and hyperactivity).	

The exclusion criteria were: (1) interventions conducted or assessed in a language other than English (2) studies including children with intellectual disabilities (Verbal IQ < 70) (3) reviews, conference proceedings, abstracts, theses, or protocols. Studies that were not conducted and assessed in English were excluded in order to reduce the possibility of changes occurring due to translations or the cultural context. Studies including children with ID were also excluded to reduce sample heterogeneity.

#### Meta-analysis

The authors of studies using the SRS and/or SSRS were contacted for missing total and subscale scores.

### Quality Assessment: Risk of Bias

Two reviewers (JW and EK) independently assessed the quality of eligible studies employing the Cochrane Collaboration Risk of Bias (RoB) v2 tool (Higgins 2016). The studies were assessed for bias in sequence generation, allocation concealment, baseline measurements, blinding or participants and personnel, blinding of outcome assessments, addressing incomplete outcomes, selective reporting and other potential biases (Higgins 2016) (Supplementary materials). Any disagreements between reviewers were resolved through discussion and consensus was reached on all ratings.

### Data Extraction

Two reviewers independently extracted data (JW and EK) using a bespoke data extraction spreadsheet. The extraction spreadsheet is available from the authors upon request. Data were extracted on the intervention characteristics, patient characteristics, parental outcome measures used, and subsequent outcome scores. Authors were contacted for additional information when necessary.

Authors were contacted to provide total scores and subscale scores of the SRS and SSRS that were not published. The co-variates were the intervention type, duration (in hours), intensity (weekly vs summer camp), teaching strategy (didactic vs performance) and whether (yes/no) there was parental involvement in the intervention.

### Data Analysis

#### Meta-analysis

Statistical analysis was conducted using STATA 14. The standardized mean difference (SMD) and 95% confidence interval for each outcome measure were used as a summary statistics. The post treatment measures of the treatment and delayed control groups were compared across studies. The SMD was interpreted as a small effect size for values of 0.20–0.50, moderate for values of 0.50–0.80, large for values of 0.80–1.30 and very large for values above 1.30 (Cohen 1988).

The random–effects model was used, as heterogeneity was suspected in the data. Heterogeneity was assessed using the Higgins heterogeneity I^2^ statistic. The degree of heterogeneity was considered low for values of 25–49%, moderate for values of 50–74% and high for values of 75% or more (Higgins et al. 2003). Statistically significant heterogeneity was assumed when p < 0.05.

#### Sensitivity Analyses

Publication bias was assessed using funnel plots with Egger’s test, and the trim and fill method (Egger et al. 1997).

## Results

### Study Selection

#### Systematic Review

**Fig. 1:**
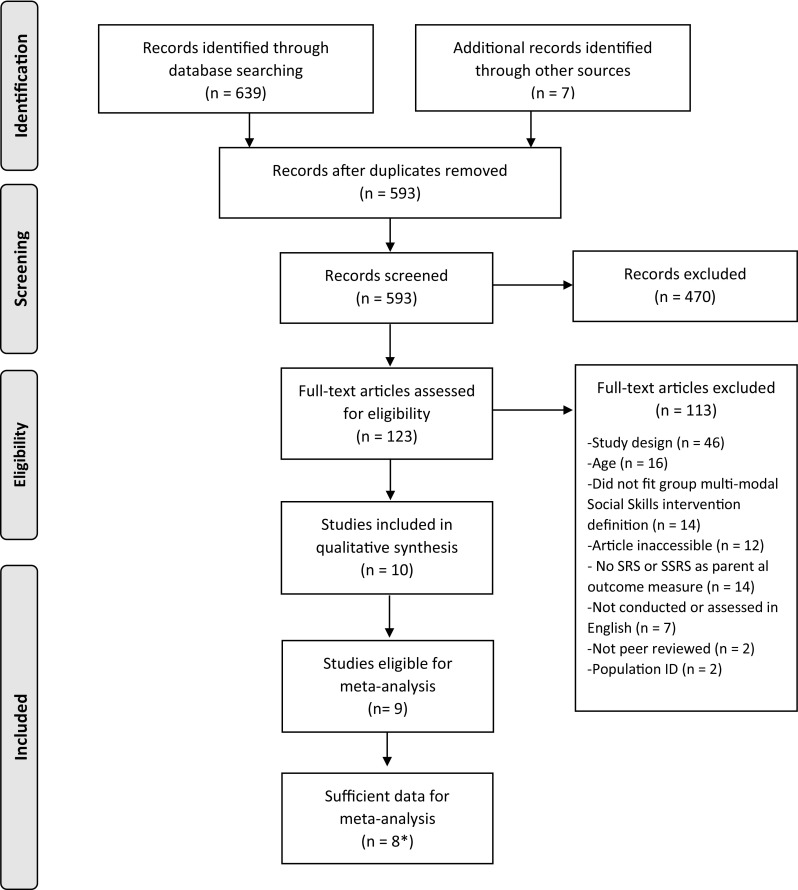
Prisma flow diagram. *Eight studies used the SRS (n = 5), the SSRS (n = 1) or both (n = 2)

The electronic search returned 593 articles after duplicates were removed. Additional articles were identified through correspondence with authors and by screening reference lists of review articles picked up in the initial screening search. Studies were excluded if they did not fit the inclusion criteria or did not fit this review’s definition of group social skills interventions (Fig. 1). The screening process reduced the number of eligible articles to 123 that were fully assessed for eligibility. 10 studies that met criteria for eligibility were retained for qualitative synthesis.

#### Meta-analysis

The use of outcome measures was assessed in the 10 studies retained for qualitative synthesis. The authors were contacted for unpublished total and subscale scores. Following this correspondence there were sufficient data to conduct meta-analyses on 8 studies (5 used the SRS, 1 used the SSRS and 2 used both the SRS and SSRS).

### Qualitative Synthesis

#### Intervention Characteristics

Five different types of intervention programmes were used, including established protocols such as PEERS, Children’s Friendship Training, summerMAX and SENSE Theatre; as well as an unnamed manualised Cognitive Behavioural Therapy (CBT) social skills programme. The programmes varied by teaching strategy, parent assistance, duration and intensity (Table 1). All but one of the programmes (SENSE Theatre) took a didactic teaching approach. SENSE theatre was the only GSSI to employ a performance teaching strategy.

**Table 1:** Intervention characteristics

Article	Intervention	M age	N	Number of sessions	Teaching Strategy	Additional input	Parent
Corbett et al. (2016)	SENSE theatre	11.27	30	240 min/10 sessions	Performance	Peer assisted	- SRS
							- ABAS
Gantman et al. (2012)	PEERS young adults	20.4	17	90 min/14 sessions	Didactic	Parent group	- SSRS
							- SRS
							- EQ
							- QSQ
Koning (2013)	Not named—CBT social skills	11.07	15	120 min/15 sessions	Didactic	Parent handout	- VABS-2
							- SRS
Laugeson et al. (2009)	PEERS	14.6	33	90 min/12 sessions	Didactic	Parent group	- SSRS
							- QPQ
Laugeson et al. (2015)	PEERS young adults	21.39*	22	90 min/16 sessions	Didactic	Parent group	- SRS
							- SSRS
							- QSQ
							- EQ
Lopata et al. (2010)	Adapted skillstreaming	9.47	36	350 min/5 days per week for 5 weeks	Didactic	Parent group	- ASC
							- SRS
							- BASC-2-PRS
							- Satisfaction survey
Schohl et al. (2014)	PEERS	13.65	58	90 min/14 sessions	Didactic	Parent group	- QSQ
							- SRS
							- SSRS
Thomeer et al. (2012)	Adapted skillstreaming	9.31	35	350 min/5 days per week for 5 weeks	Didactic	Parent group	- ASC
							- SRS
							- BASC-2-PRS
							- Satisfaction survey
Thomeer et al. (2016)	summerMAX	9.15	57	350 min/5 days per week for 5 weeks	Didactic	Parent group	- ASC
							- SRS-2
							- BASC-2-PRS
							- Satisfaction survey
Waugh and Peskin (2015)	SSToM CFT	9	49	SSToM: not disclosed CFT: 60 min/10 weekly sessions	Didactic Didactic	Parent group Parent group	- SRS-2

Interventions**—***CFT* children’s friendship training, *PEERS* program for the education and enrichment of relational skills, *SENSE theatre* SENSE theatre, *SSToM* social skills and theory of mind

Parent outcome measures**—***ABAS* adaptive behaviour assessment schedule, *ASC* adapted skillstreaming checklist, *BASC-2- PRS* behavior assessment system for children–parent rating scales, second edition, *EQ* empathy quotient, *QSQ* quality of socialisation questionnaire, *QPQ* quality of play questionnaire, *SRS* social responsiveness scale, *SSRS* social skills rating scale, *VABS-2* vineland adaptive behaviour system, second edition

All GSSIs ran children groups, most interventions also ran parallel parent groups. Only the SENSE Theatre and the unnamed CBT social skills programme did not run parent groups (the CBT intervention did provide a handout for parents). The summerMAX and the SENSE Theatre programmes ran intense summer-camp style interventions where participants were required to attend 4–5 h of training 5 days a week for 2–5 weeks. The other programmes were less intensive and comprised 60–90 min sessions once a week for 10–16 weeks.

The syllabuses of GSSIs varied. Each GSSI emphasised different domains of social skills. These included social knowledge, social communication, social cognition and social emotions. Specifically, the interventions taught social rules and social cues, pragmatic language skills, cognitive social skills including problem solving, cognitive flexibility, social perception and/or perspective taking. All but PEERS taught non-verbal skills, such as social eye contact, facial expression, posture and social distance. Only the summerMAX programme focussed explicitly on self-perception (e.g. understanding one’s own emotions). Only SENSE theatre and PEERS addressed the issue of affect regulation (e.g. how to be a good sport, controlling emotional impulses or anxiety).

#### Assessment Characteristics

Although the programmes selected for this meta-analysis must have employed the SRS/SSRS, other parent-rated measures included the adapted skillstreaming checklist (ASC), the empathy (EQ) and the behavior assessment system for children–parent rating scales (BASC-PRS-2) (Table 2). We have not examined the psychometric properties of any of these assessment instruments in detail (see Cordier et al. 2015; Matson and Wilkins 2009 for comprehensive reviews).

**Table 2:** Assessments by informant type

Article	Parent questionnaire	Self-report questionnaire	Task	Teacher questionnaire	Staff/observation
Corbett et al. (2016)	- SRS - ABAS		- NEPSY - ERP incidental face memory task		- Peer interaction paradigm
Gantman et al. (2012)	- SSRS - SRS - EQ - QSQ	- SELSA - QSQ - TYASSK - SSI			
Koning (2013)	- VABS-2 - SRS	- Social knowledge	- CASP		- Peer interaction measure - Verbal and nonverbal behaviors coding
Laugeson et al. (2009)	- SSRS - QPQ	- QPQ - TASSK - FQS		- SSRS	
Laugeson et al. (2015)	- SRS - SSRS - QSQ - EQ	- QSQ - TYASSK			
Lopata et al. (2010)	- ASC - SRS - BASC-2- PRS - Satisfaction survey	- Satisfaction survey	- DANVA-2 - CASL idioms - SKA		- Satisfaction survey - ASC - SRS - BASC-2-TRS
Schohl et al. (2014)	- QSQ - SRS - SSRS	- TASSK - QSQ - FQS - SIAS		- SRS - SSRS	
Thomeer et al. (2012)	- ASC - SRS - BASC-2-PRS - Satisfaction Survey	- Satisfaction survey	- DANVA-2 - CASL idioms - SKA		- Satisfaction survey - ASC - SRS - BASC-2-TRS
Thomeer et al. (2016)	- ASC - SRS-2 - BASC-2-PRS - Satisfaction survey	- Satisfaction survey	- CASL idioms		- Satisfaction survey - ASC - SRS 2 - BASC-2-TRS
Waugh and Peskin (2015)	- SRS-2		- Revised version of the strange stories test - Theory of mind inventory		

Outcome measures—*ABAS* adaptive behaviour assessment schedule, *ASC* adapted skillstreaming checklist, *BASC-2-PRS* behavior assessment system for children–parent rating scales, second edition, *BASC- 2-TRS* behavior assessment system for children–teacher rating scales, second edition, *CASL* comprehensive assessment of spoken language, *CASP* child and adolescent social perception measure, *EQ* empathy quotient, *DANVA-2* diagnostic analysis of nonverbal accuracy2, *FQS* friendship qualities scale, *NEPSY* developmental neuropsychological assessment, *QSQ* quality of socialisation questionnaire, *QPQ* quality of play questionnaire, *SELSA* social and emotional loneliness scale for adults, *SIAS* social interaction anxiety scale, *SKA*: skillstreaming knowledge assessment, *SRS* social responsiveness scale, *SSI* social skills inventory, *SSRS* social skills rating scale, *TASSK* test of adolescent social skills knowledge, *TYASSK* test of young adult social skills knowledge, *VABS-2* vineland adaptive behaviour system, second edition

All of the studies retained for qualitative synthesis used more than one type of informant, not only parents but also the participants themselves, study staff and teachers (Table 2). Two studies reported only on questionnaires completed by parents and participants; five used socio-cognitive tasks and three used an idiomatic language task with participants. Four used self-report questionnaires in conjunction with a socio-cognitive or idiomatic language task. None used validated self-report questionnaires in conjunction with socio-cognitive tasks; participants are best placed to report on changes in their social knowledge, implying the GSSI studies reviewed here may not be capturing changes in this social skills dimension.

Two studies used teacher-report measures (SRS and SSRS). Two also used observation schedules to measure social performance. Participants were filmed interacting with confederate peers, one was blind-rated. The studies that used staff questionnaires administered satisfaction surveys that were not validated; the questionnaires were completed by non-blind observers.

### Quality Assessment: Risk of Bias

A ‘risk of bias’ analysis was conducted on all the RCTs (Table 3). Two studies obtained a ‘high risk’ rating in four or more of the seven risk of bias criteria; these will be discussed separately. All others obtained a ‘low risk’ or ‘unclear’ rating for the *sequence generation* and *allocation concealment* criteria. The incomplete blinding of outcome by participants, personnel and outcome assessors conferred a ‘high risk’ for all of the studies. A few studies did employ observational outcome measures (where the coders were blind to the participants’ group status) but these were always used in conjunction with outcome measures where the assessors were not blind. The incomplete-outcome criteria were rated ‘high risk’ for two-thirds of the studies, because of participant attrition from either or both the waitlist control and the intervention groups. The selective-outcome reporting criterion was rated ‘low risk’ in all studies. No other sources of bias were detected.

**Table 3:** Risk of bias assessment

RCTS	Sequence generation	Allocation concealment	Baseline measurements	Blinding of participants and personnel	Blinding of outcome assessors	Incomplete outcome data	Selective outcome reporting
Corbett et al. (2016)	Low risk	Low risk	High risk	High risk	High risk	High risk	Low risk
Gantman et al. (2012)	Low risk	Unclear	Low risk	High risk	High risk	Low risk	Low risk
Koning (2013)	Low risk	Low risk	Low risk	High risk	High risk	High risk	Low risk
Laugeson et al. (2009)	Unclear	Unclear	Low risk	High risk	High risk	High risk	Low risk
Laugeson et al. (2015)	Low risk	Low risk	Low risk	High risk	High risk	High risk	Low risk
Lopata et al. (2010)	Low risk	Unclear	Low risk	High risk	High Risk	Low risk	Low risk
Schohl et al. (2014)	Unclear	Unclear	Low risk	High risk	High risk	High Risk	Low risk
Thomeer et al. (2012)	Unclear	Unclear	Unclear	High risk	High risk	Low risk	Low risk
Thomeer et al. (2016)	Low risk	Unclear	Low risk	High risk	High risk	Low risk	Low risk
Waugh and Peskin (2015)	High risk	High risk	High risk	High risk	High risk	High risk	Low risk

Two studies, (Corbett et al. 2016; Waugh and Peskin 2015) obtained more ‘high risk’ ratings than others reviewed here. The Waugh and Peskin (2015) study scored ‘high risk’ for all except selective-outcome reporting criteria. The baseline measures were ‘high risk’ because SRS scores differed significantly at baseline between the control and experimental groups, and this study was excluded from the meta-analysis. The Corbett study obtained a ‘high risk’ rating for the baseline measurements criteria due to a discrepancy between control and experimental groups on two outcome measures (theory of mind and delayed faces memory). As this baseline discrepancy did not affect the SRS or SSRS scores, the Corbett study was retained for analysis.

### Meta-analysis

#### Social Responsiveness Scale (SRS)

**Fig. 2:**
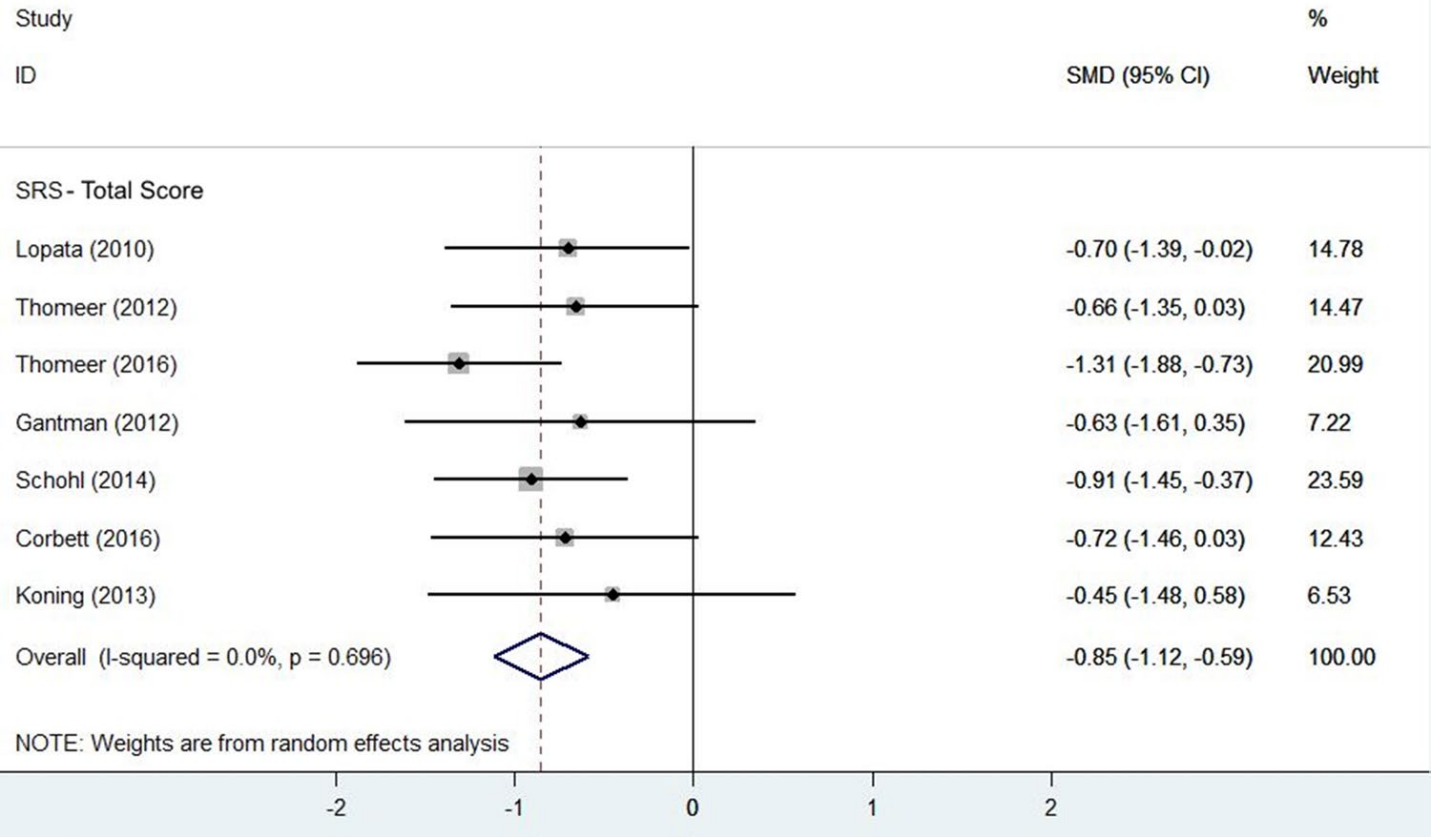
Forest plot of SRS total scores

A comparison of the treatment and control groups’ post-intervention scores showed GSSI participants obtained better outcomes than controls, with a substantial reduction in SRS total scores (SMD = − 0.85, 95% CI [− 1.12,− 0.59], Z = 6.35, p = 0.000; Fig. 2; Table 4). This is a significant (p < 0.0001) and large effect size.

**Table 4:** Meta-analysis summary table

Study	n		SRS Total score	SSRS Social skills	SSRS Problem behaviours
	T	WLC	SMD (95% CI)	SMD (95% CI)	SMD (95% CI)
Corbett et al. (2016)	17	13	− 0.72 (− 1.46, 0.03)	–	–
Koning (2013)	7	8	− 0.45 (− 1.48, 0.58)	–	–
Lopata et al. (2010)	18	17	− 0.7 (− 1.39, − 0.02)	–	–
Thomeer et al. (2012)	17	17	− 0.66 (− 1.35, 0.03)	–	–
Thomeer et al. (2016)	28	29	− 1.31 (− 1.88, − 0.73)	–	–
Gantman et al. (2012)	9	8	− 0.63 (− 1.61, 0.35)	0.47 (− 0.50, 1.44)	− 0.11 (− 1.06, 0.84)
Schohl et al. (2014)	29	29	− 0.91 (− 1.45, − 0.37)	0.45 (− 0.07, 0.97)	− 0.35 (− 0.36, 0.17)
Laugeson et al. (2009)	17	16	–	0.83 (0.12, 1.54)	− 1.15(− 1.89, − 0.41)
Total			− **0.85** (− 1.12, − 0.59)**	**0.56** (0.18, 0.95)*	− **0.55** (− 1.13, 0.03)

Laugeson 2015 data is not presented in this table as we were not able to gain access to the primary data

*T* treatment, *WLC* waitlist control

*p < 0.05

**p < 0.0001

GSSI participants also improved on all SRS subscales, relative to controls (Table 5). The effect sizes for the social awareness (SMD = − 0.57, 95% CI [− 0.87,− 0.28], Z = 3.78, p = 0.000), social cognition (SMD = − 0.53, 95% CI [− 0.98,− 0.09], Z = 2.34, p = 0.019) and social motivation subscales (SMD = − 0.55, 95% CI [− 1.02,− 0.07], Z = 2.27, p = 0.023) were moderate. The effect sizes on the social communication (SMD = − 0.89, 95% CI [− 1.2,− 0.59], Z = 5.71, p = 0.000) and restricted interests and repetitive behaviours subscales (SMD = − 0.9, 95% CI [− 1.23,− 0.57], Z = 5.4, p = 0.000) were large. All subscale effect sizes were significant (p < 0.05).

**Table 5:** Meta-analysis SRS total score and subscale effect sizes

SRS		n		Total score	Social awareness	Social cognition	Social communication	Social motivation	Restricted interests and repetitive behaviour
Study	Intervention	T	WLC	SMD (95% CI)	SMD (95% CI)	SMD (95% CI)	SMD (95% CI)	SMD (95% CI)	SMD (95% CI)
Corbett et al. (2016)	SENSE Theatre	17	13	− 0.72 (− 1.46, 0.03)	− 0.26 (− 0.99, 0.46)	− 0.6 (− 1.34, 0.14)	− 0.89 (− 1.65, − 0.13)	− 0.24 (− 0.96, 0.49)	− 0.49 (− 1.22, 0.25)
Koning (2013)	Not named – CBT Social Skills	7	8	− 0.45 (− 1.48, 0.58)	− 0.45 (− 1.48, 0.58)	0.32 (− 0.70, 1.34)	− 0.53 (− 1.56, 0.51)	− 0.14 (− 1.16, 0.87)	− 0.85 (− 1.91, 0.22)
Lopata et al. (2010)	summerMAX	18	17	− 0.7 (− 1.39, − 0.02)	− 0.31 (− 0.98, 0.36)	− 0.23 (− 0.89, 0.44)	− 0.76 (− 1.45, − 0.07)	− 0.96 (− 1.67, − 0.26)	− 0.51 (− 1.19, 0.16)
Thomeer et al. (2012)	summerMAX	17	17	− 0.66 (− 1.35, 0.03)	− 0.4 (− 1.08, 0.28)	− 0.43 (− 1.11, 0.25)	− 0.59 (− 1.28, 0.10)	− 0.24 (− 0.91, 0.44)	− 1.04 (− 1.76, − 0.32)
Thomeer et al. (2016)	summerMAX	28	29	− 1.31 (− 1.88, − 0.73)	− 1.1 (− 1.66, − 0.54)	− 1.33 (− 1.90, − 0.75)	− 1.44 (− 2.03, − 0.86)	− 1.35 (− 1.93, − 0.77)	− 1.42 (− 2.00, − 0.84)
Gantman et al. (2012)	PEERS	9	8	− 0.63 (− 1.61, 0.35)	− 0.57 (− 1.55, 0.40)	− 0.54 (− 1.51, 0.44)	− 0.6 (− 1.58, 0.38)	0.02 (− 0.93, 0.97)	− 0.87 (− 1.87, 0.13)
Schohl et al. (2014)	PEERS	29	29	− 0.91 (− 1.45, − 0.37)	–	–	–	–	–
Total		125	121	− **0.85** (− 1.12, − 0.59)**	**− 0.57** (− 0.87, − 0.28) **	− **0.53** (− 0.98, − 0.09) *	− **0.89** (− 1.2,− 0.59) **	− **0.55** (− 1.02, − 0.07)*	− **0.9** (− 1.23, − 0.57)**

*T* treatment, *WLC* waitlist control

*p < 0.05

**p < 0.0001

**Fig. 3:**
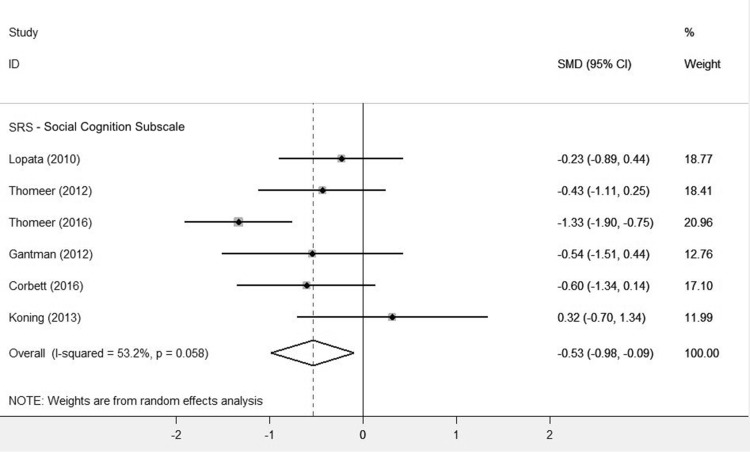
Forest plot of SRS social cognition subscale scores. Schohl et al. 2014 cognition subscales were not included in the analysis as the source data was not available

Koning et al. (2013; Fig. 3) was the only study not to report improvement in the social cognition subscale.

#### Social Skills Rating System (SSRS)

**Fig. 4:**
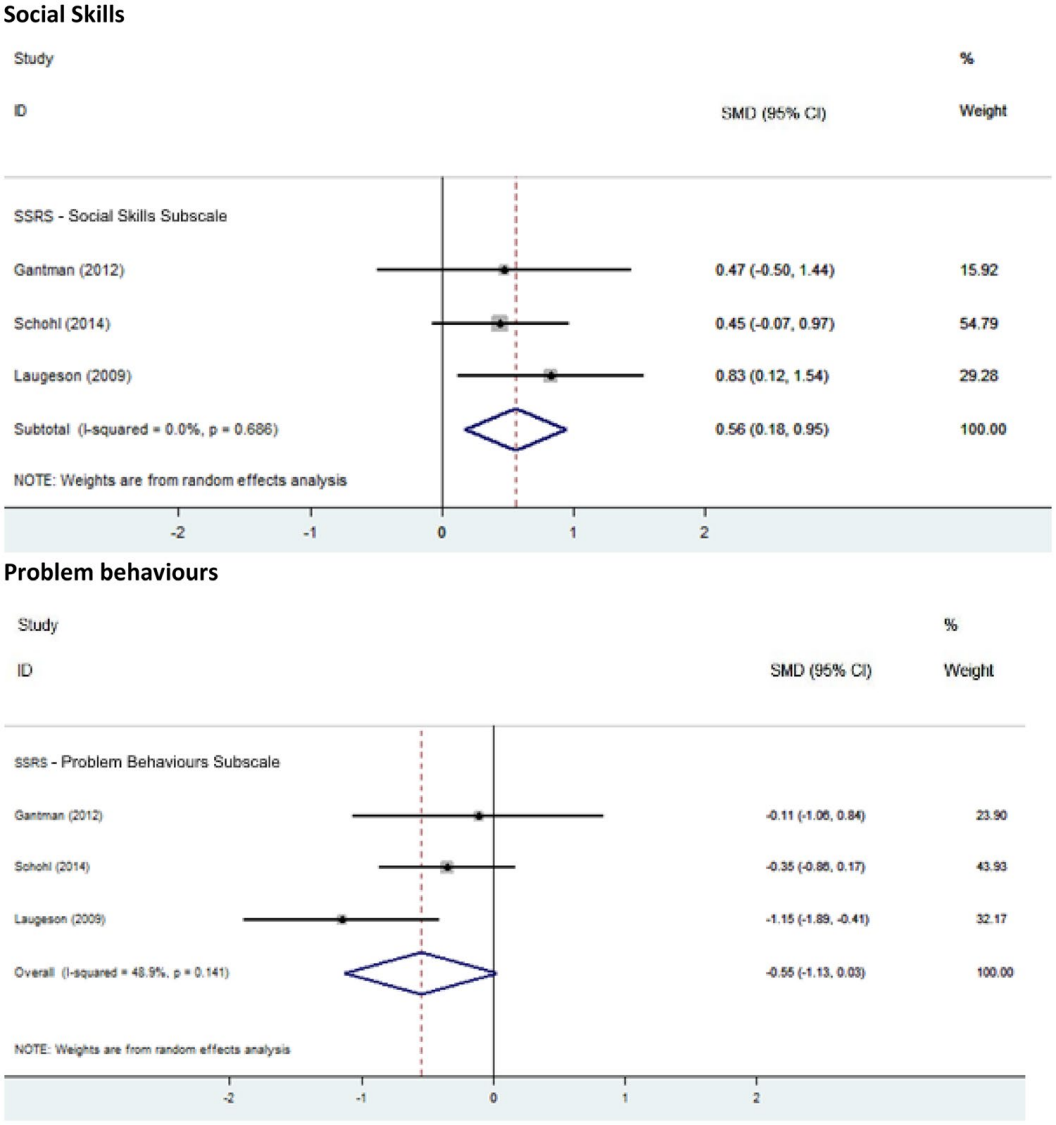
Forest plot of SSRS social skills and problem behaviours subscale scores

GSSI participants improved relative to controls on the social skills subscale (SMD = 0.56, 95% CI [0.18,0.95], Z = 2.86, p = 0.004) and had better outcomes on the problem behaviours subscale (SMD = − 0.55, 95% CI [− 1.13,0.03], Z = 1.86, p = 0.06; Fig. 4). The effect size for both subscales was moderate, but only the social skills subscale effect was significant.

### Moderator Analysis

Moderator analyses was conducted on the SRS. There were insufficient studies to conduct moderator analyses on the SSRS.

#### SRS Group Analysis by Intervention

**Fig. 5:**
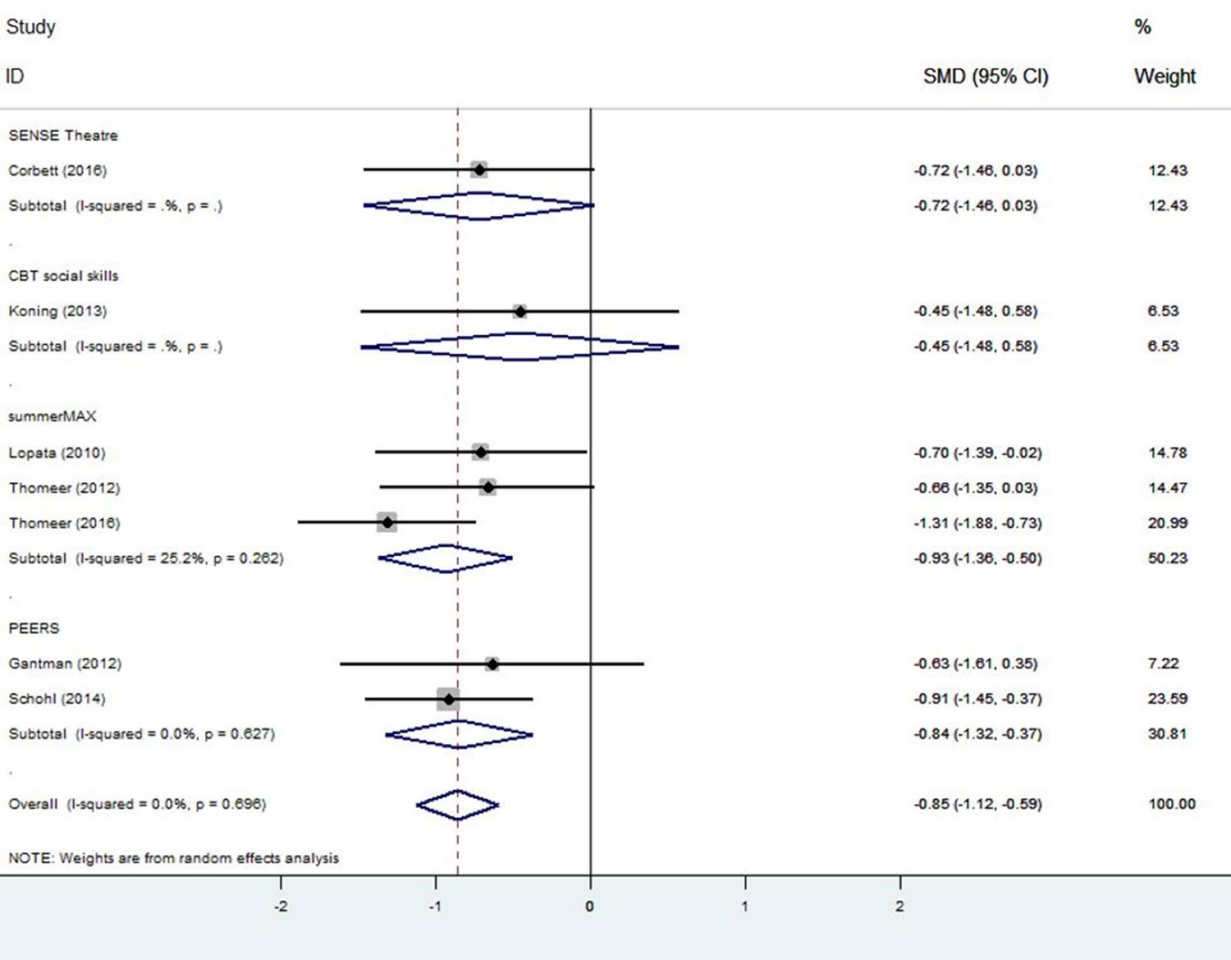
Group analyses forest plot by intervention programme for the SRS total scores

A post-hoc analysis analysed group differences on the total SRS scores by separating studies according to intervention type (Fig. 5). There was no statistical difference in the total SRS scores between the treatment and control group for the SENSE theatre (p = 0.06) or the CBT social skills intervention (p = 0.39), but sample size was small so there was a potential Type II error. The SENSE theatre intervention obtained a moderate effect size (SMD = − 0.72, 95% CI [− 1.46,0.03], Z = 1.88); the CBT intervention had a small effect size (SMD = − 0.45, 95% CI [− 1.48,0.58], Z = 0.86).

summerMAX was used in 3 studies and PEERS was used in 2 studies. Participants receiving these interventions obtained better outcomes than controls (p < 0.0001). Both summerMAX (SMD = − 0.93, 95% CI [− 1.36,− 0.5], Z = 4.22) and PEERS (SMD = − 0.84, 95% CI [− 1.32,− 0.37], Z = 3.49) obtained large and significant effect sizes.

#### SRS Group Analysis by Parent Involvement

**Fig. 6:**
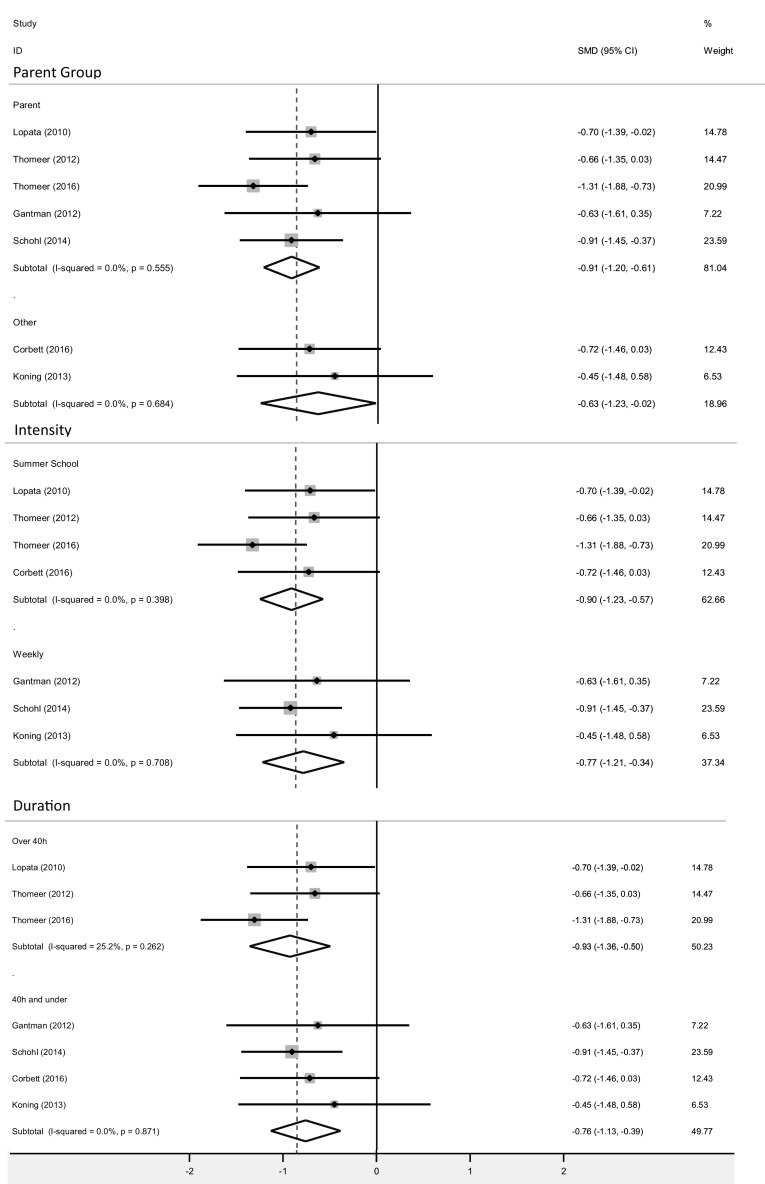
Group analyses forest plot for parent involvement (parent group vs no parent group), intervention intensity (summer school vs weekly) and intervention duration (over 40 vs 40 h and under) for the SRS total scores

A group analysis was conducted on the total SRS score according to parent involvement. Participants performed better than controls regardless of whether they took part in an intervention that delivered concurrent parent groups, both effect sizes were significant (parent group p < 0.0001; no parent group p = 0.04). The GSSIs that delivered parent groups had a large effect size (SMD = − 0.91, 95% CI [− 1.20,− 0.61], Z = 6.08) whereas the GSSI that did not deliver parent groups had a moderate effect size (SMD = − 0.63, 95% CI [− 1.23,− 0.02], Z = 2.03; Fig. 6).

#### SRS Group Analysis by Intensity and Duration

Group analyses were conducted for the intensity and duration of GSSIs on total SRS scores (Fig. 6). The effect sizes in both the intensity and duration group analyses were significant (p < 0.0001). The more intensive GSSIs which took a summer camp format had a large effect size (SMD = − 0.90, 95% CI [− 1.23,− 0.57], Z = 5.3), whereas the GSSI taking place once a week had a moderate effect size (SMD = − 0.77, 95% CI [− 1.21,− 0.34], Z = 3.35).

GSSIs groups to examine the effect of duration of intervention as a co-variate were created with a median split. The GSSIs which required over 40 h of contact time also had a large effect size (SMD_> 40 h_ = − 0.93, 95% CI [− 1.36,− 0.50], Z = 4.22), whereas those requiring 40 h and under had a moderate effect size (SMD_< 40 h_ = − 0.76, 95% CI [− 1.13,− 0.39], Z = 4.00; Fig. 6).

#### Heterogeneity

Heterogeneity was assessed using the I^2^ statistic. The heterogeneity in the data was low to moderate, ranging from 0 to 58.2%. However, results did not differ across random and fixed effect models.

#### Publication Bias

Egger’s regression test and the trim and fill method showed that there was no evidence of substantial publication bias.

## Discussion

Our systematic review of RCTs using multi-modal GSSIs has shown that studies use a variety of social skills measures, assessment types and informants. There was a predominant reliance on parent-report and self-report assessments of effectiveness, both prone to expectancy bias. Even when evidence of outcome was obtained from external observers such as support staff or teachers, these observers were seldom blind to treatment group. In future, evaluations of GSSI should employ blind-rated observer-reports (of performance). There is currently a lack of validated participant self-reports (of increase in social skills knowledge), yet previous meta-analyses of social knowledge improvement indicate this may be one of the main gains from group social skills interventions (Gates et al. 2017).

Evidence of the effectiveness of interventions from the meta-analysis of the SRS indicated treatments do bring about a significant reduction in autistic traits as measured by total and subscale scores, by parental report. Large effect sizes were found in terms of improved Social Communication, and reduced Restricted Interests and Repetitive Behaviour (RRB). The Social Communication scale of the SRS is intended to capture ‘expressive social communication [and] “motoric” aspects of reciprocal social behaviour’ (Constantino and Gruber 2012). Both subscales were derived from clinical definitions, rather than factor analysis, and reflect the main components of DSM-5 diagnostic criteria for Autism Spectrum Disorders.

Moderate effect sizes for improvement following intervention, explicitly in terms of social skills, were found for the Social Skills subscale of the SSRS, which measures cooperation, empathy, assertion, self-control and responsibility. Unfortunately, there were insufficient data available to enable further analysis of the Social Skills subscale, as it would have been interesting to see which items contributed the most to the significant changes in behaviour. The Problem Behaviours subscale of the SSRS measures internalising and externalising behaviours, and hyperactivity; no significant change was found in these behaviours.

Despite the differences in the social skills domains taught in GSSIs, the syllabuses did overlap in some key areas. For instance, they all aimed to improve social communication skills, and evidence from this review that Social Communication does improve significantly could have been anticipated. However, improvements on the RRB subscale of the SRS were unexpected; no teaching materials reviewed here explicitly target RRB. Perhaps the cognitive and emotional skills taught during GSSIs, such as cognitive flexibility, problem solving or controlling emotional impulses are mediating this change. Consequently, participants become more confident and less anxious in social situations, which in turn reduces their anxiety-related restrictive and repetitive behaviours (Rodgers et al. 2012). Also, participants may learn that restrictive and repetitive behaviours are socially inappropriate, and consequently they conceal them, a hypothesis that is consistent with the moderate effect size obtained on the Social Awareness subscale. Evidence from previous meta-analyses of GSSI shows increases in social knowledge drive effect sizes in self-report measures of social skills (Gates et al. 2017).

Moderator analysis was only possible for studies in which the SRS was the outcome measure. A group analysis compared interventions that delivered concurrent parent groups, with those that did not. We found that GSSIs that included parent groups were more effective, associated with a large (compared with a moderate) effect size. Parents who attend GSSIs might display positive response biases (McMahon, Lerner et al., 2013), but parent involvement in treatment can nevertheless consolidate the social behaviours and knowledge acquired by their child, and help support the formation of appropriate peer networks (Laugeson and Frankel 2011).

Not all GSSI programmes reduced autistic traits (as measured by SRS total scores). The PEERS and summerMAX programmes obtained significant and large effect sizes compared to the SENSE Theatre and CBT social skills interventions (though associated with less power to detect benefit) which obtained small to moderate and non-significant effects effect sizes.

More intensive and longer-lasting interventions had slightly larger effect sizes. The cost-benefit comparison between programmes is hard to interpret. For instance, whereas the PEERS intervention is demanding in terms of participant and interventionist time, it may nevertheless be a more cost-effective choice as it is easier to implement with less resources than the summerMAX programme. Only one out of the six interventions employed a performance-based teaching strategy, therefore a comparison between didactic and performance based interventions was not possible.

## Conclusion

A recent increase in methodological rigour in GSSI RCTs, and the use of common instruments to assess outcomes, has presented an opportunity to examine the effectiveness of social-skills interventions in a multi-dimensional context. Understanding what works for whom will be key to the future personalisation of GSSIs, improving the efficacy of GSSI programmes. Examining which social performance and social knowledge characteristics are responsive to specific GSSI design features is critical to unlocking our understanding of the active ingredients of social skills instruction. We need to develop more sensitive tools in order comprehensively to capture how treatments impact on the multi-dimensional nature of social skills.
